# Sex Differences in Vitamin D Status as a Risk Factor for Incidence of Disability in Instrumental Activities of Daily Living: Evidence from the ELSA Cohort Study

**DOI:** 10.3390/nu14102012

**Published:** 2022-05-11

**Authors:** Mariane Marques Luiz, Roberta de Oliveira Máximo, Dayane Capra de Oliveira, Paula Camila Ramírez, Aline Fernanda de Souza, Maicon Luís Bicigo Delinocente, Andrew Steptoe, Cesar de Oliveira, Tiago da Silva Alexandre

**Affiliations:** 1 Postgraduate Program in Physical Therapy, Federal University of Sao Carlos, Rodovia Washington Luís, Km 235, SP-310, Sao Paulo 13565-905, Sao Carlos, Brazil; mariane_marques@hotmail.com (M.M.L.); bhetamx@hotmail.com (R.d.O.M.); dayacapra@gmail.com (D.C.d.O.); paulacamilaramirez@gmail.com (P.C.R.); linefernandasouza@gmail.com (A.F.d.S.); 2 School of Physical Therapy, Santander Industrial University, Cra 27, Calle 9, Santander, Bucaramanga 680006, Colombia; 3 Postgraduate Program in Gerontology, Federal University of Sao Carlos, Rodovia Washington Luís, Km 235, SP-310, Sao Paulo 13565-905, Sao Carlos, Brazil; maicontema@gmail.com; 4 Department of Epidemiology and Public Health, University College London, Gower Street, London WC1E 6BT, UK; a.steptoe@ucl.ac.uk (A.S.); c.oliveira@ucl.ac.uk (C.d.O.); 5 Gerontology Department, Federal University of Sao Carlos, Rodovia Washington Luís, Km 235, SP-310, Sao Paulo 13565-905, Sao Carlos, Brazil

**Keywords:** aging, disability, incidence, instrumental activities of daily living, serum 25(OH)D concentrations, vitamin D, 25-hydroxyvitamin D

## Abstract

Vitamin D deficiency compromises elements underlying the disability process; however, there is no evidence demonstrating the association between vitamin D deficiency and the incidence of disability in instrumental activities of daily living (IADL). We investigated the association between vitamin D deficiency and the risk of incidence of IADL disability separately in men and women. A total of 4768 individuals aged ≥50 years from the English Longitudinal Study of Aging (ELSA) and without IADL disability according to the Lawton scale were available. Vitamin D was evaluated at baseline by serum 25(OH)D concentrations and classified as sufficient (>50 nmol/L), insufficient (>30 to ≤50 nmol/L) or deficient serum (≤30 nmol/L). IADL were reassessed after 4 years. Poisson models stratified by sex and controlled by covariates demonstrated that deficient serum 25(OH)D was a risk factor for the incidence of IADL disability in men (IRR: 1.43; 95% CI 1.02, 2.00), but not in women (IRR: 1.23; 95% CI 0.94, 1.62). Men appear to be more susceptible to the effect of vitamin D deficiency on the incidence of IADL disability, demonstrating the importance of early clinical investigation of serum 25(OH)D concentrations to prevent the onset of disability.

## 1. Introduction

The maintenance of independence in instrumental activities of daily living (IADL) is fundamental to avoid social isolation and assure the autonomy of older individuals [1]. Several risk factors for the incidence of IADL disability are already known—for example, advanced age, sedentary lifestyle, presence of comorbidities, depression, low visual and hearing perception, and mainly, impairment in lower limb function, reduced muscle strength and cognitive decline [1,2]. Furthermore, undernutrition, an unbalanced diet and nutritional deficits, such as vitamin D deficiency, can also decrease physiological reserves and favor highly disabling clinical conditions [3].

In this context, vitamin D deficiency, defined by serum concentrations of 25-hydroxyvitamin D (25(OH)D) ≤ 30 nmol/L, represents a global public health problem, and older adults are an age group that requires special attention [4,5]. The thinning of the epidermis as age advances results in a lower capacity for the skin to synthesize vitamin D [6,7]. These individuals also tend to spend less time outdoors in sunlight and may use medications that interfere with vitamin D metabolism. Not least, they can experience a process of malabsorption and changes in dietary patterns that lead to a decrease in food quality, quantity, and variety [8]. Thus, the consumption of the few dietary sources of vitamin D (mushrooms, tuna, salmon, cod liver oil and egg yolks) is reduced [9], which makes them more vulnerable to serum 25(OH)D deficiency.

Therefore, there is evidence of an association between low serum 25(OH)D concentrations and disability in older adults [10,11], due to the role of 25(OH)D in important components that maintain the integrity of functional capacity, such as the musculoskeletal (MSK) system and central nervous system (CNS). 25(OH)D modulates the differentiation and proliferation of muscle cells and maintains an adequate muscle metabolism [12]. Thus, its deficiency can favor a reduction in muscle strength, mass, and function [13]. Furthermore, 25(OH)D has a protective effect on neurons and glial cells, and therefore, its deficiency may predispose to cognitive decline [14].

Just as functional decline develops differently between men and women [15], the age-related decline in serum 25(OH)D concentrations is not linear between sexes, occurring first in women around the age of 50 years, and later in men around the age of 70 years [16]. Given this and the impact of 25(OH)D on MSK and cognitive function, it is plausible that deficient serum 25(OH)D can mediate the mechanism of incidence of IADL disability differently between sexes as age advances.

Cross-sectional evidence has already shown that older adults with deficient serum 25(OH)D have a higher IADL impairment score compared to individuals with serum sufficiency [17,18]. In parallel, a longitudinal study showed that older women with deficient serum 25(OH)D at baseline had a greater functional decline in IADL at 2 years of follow-up [19]. However, another study with 5 years of follow-up demonstrated that low serum 25(OH)D does not influence the trajectories of disability in very old adults in fully adjusted models [20].

Nevertheless, in addition to presenting discrepant results, none of these studies investigates the association between vitamin D status and the risk of incidence of IADL disability and how it occurs in men and women. Therefore, our objective was to investigate whether there is an association between deficient serum 25(OH)D and the risk of incidence of disability in IADL separately in English men and women.

## 2. Materials and Methods

### 2.1. Study Population

The English Longitudinal Study of Ageing (ELSA) is an ongoing prospective observational study of community-dwelling English individuals aged ≥50 years. The ELSA sample was drawn from individuals who, in 1998, 1999 and 2001, participated in the Health Survey for England (HSE), an annual health examination survey, which recruits a nationally representative sample using a multi-staged stratified random probability design [21].

The ELSA study began in 2002/2003 (Wave 1). Data are collected biannually using computer-assisted personal interviews and self-completion questionnaires. ELSA collects information on people’s physical and mental health, wellbeing, finances, and attitudes around aging and how these change over time. As of 2004/2005 (Wave 2), health examinations began to be performed through visits by a qualified nurse team and were repeated every 4 years to assess biomarkers, anthropometric measures, and physical performance. More information on the study design and sampling procedures can be found elsewhere [22]. The ELSA Study has been approved by The National Research Ethics Service (London Multicentre Research Ethics Committee [MREC 01/2/91]), and all participants gave written informed consent. This study has been conducted in accordance with all relevant ethical regulations.

**Figure 1:**
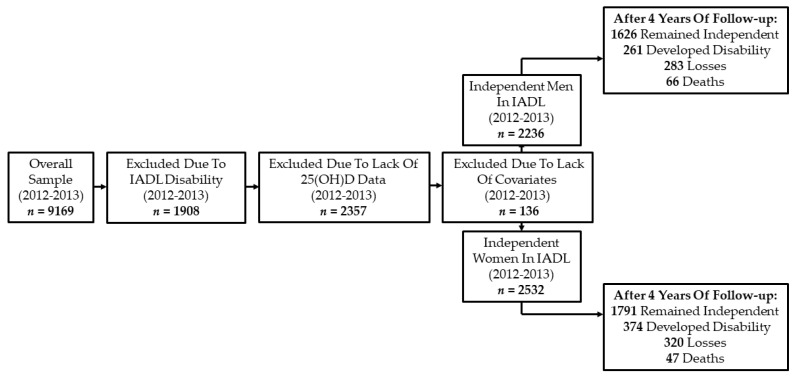
Sample selection flowchart (2012/2013–2016/2017). IADL: instrumental activities of daily living; 25(OH)D: 25-hydroxyvitamin D.

In the ELSA study, serum 25(OH)D concentrations were collected for the first time in 2012–2013 (Wave 6), which corresponded to the baseline of this study. This wave was composed of 9169 participants, of whom 1908 were excluded due to having disability in IADL, 2357 due to lack of data on serum 25(OH)D concentration, and 136 due to lack of data on the covariates used in this study [23]. Thus, the final analytical sample consisted of 4768 participants (2236 men and 2532 women), who were followed up for 4 years, and the outcome disability in IADL was measured in 2016–2017 (Wave 8). The sample selection process is shown in Figure 1.

### 2.2. Measurement of Outcome

According to the adapted Lawton scale, the following IADL were considered: preparing meals, shopping, using transportation, housekeeping, using the telephone, handling finances, and managing medications [24]. Self-reported difficulty in the seven activities was investigated at baseline and reassessed after 4 years of follow-up. Only individuals without difficulty in performing any IADL at baseline were included. The IADL outcome was defined: “remained independent for all IADL during the follow-up period” or “developed difficulty to perform one or more IADL during the follow-up period”. 

### 2.3. Measurement of Exposure

Serum 25(OH)D concentrations were analyzed in the Royal Victoria Infirmary (Newcastle upon Tyne, United Kingdom) by DiaSorin Liaison immunoassay, which provides the total circulating 25(OH)D concentrations and has an analytical sensitivity of 7.5 nmol/L, with a variation coefficient ranging from 8.7% to 9.4%. All assays were performed in duplicate, and the laboratory that performed the 25(OH)D analyses participated in the Vitamin D External Quality Assessment Schemes (DEQAS) [25]. According to the Institute of Medicine, serum 25(OH)D concentrations were classified as: sufficient (>50 nmol/L), insufficient (>30 and ≤50 nmol/L) and deficient (≤30 nmol/L) [26].

### 2.4. Covariates

Covariates were measured at baseline and selected based on previous studies involving serum 25(OH)D deficiency and functional disability. 

The socioeconomic characteristics included age (grouped into five 10-year categories as 50–59; 60–69; 70–79; 80–89 and 90 or more), race (white and non-white), conjugal life (with or without), schooling (>13 years; 12–13 years; ≤11 years) and total household wealth classified in quintiles [25].

The health-related behaviors included were smoking status (non-smoker, former smoker, or current smoker); frequency of alcohol consumption (rarely or never if 1 day a week, frequently if 2–6 days a week or daily). Based on an instrument validated by the HAS to assess the level of physical activity [27], a participant was considered active (vigorous or moderate physical activity more than once a week), or sedentary (vigorous or moderate physical activity once per week, one to three times per month, hardly ever or never; any mild physical activity) [25].

Health conditions were obtained through self-reports of medical diagnosis of systemic arterial hypertension, diabetes mellitus, cancer, lung disease, heart disease, stroke, osteoporosis, osteoarthritis, dementia and falls in the last year. Depressive symptoms were considered using the shortened version of The Center for Epidemiologic Studies Depression Scale (CES-D) (cut-off ≥ 4) [28]. 

Memory was assessed by a list of 10 words, in which participants had to read and immediately repeat the words they remembered, to assess immediate memory, and repeat after five minutes, to assess delayed memory. The correctly remembered words were summed (range 0–20), and higher scores corresponded to better memory performance [29].

Waist circumference (WC) was used to measure abdominal obesity (WC > 102 cm for men and >88 cm for women). Body mass index (BMI) calculated using the standard formula (kg/m^2^) was used to classify individuals as underweight (<18.5 kg/m^2^), ideal weight (≥18.5 and <25 kg/m^2^), overweight (≥25 and <30 kg/m^2^) and obese (≥30 kg/m^2^) [30]. Handgrip strength was used to diagnose dynapenia (<26 kg for men and <16 kg for women) [31].

The seasons of collected serum 25(OH)D concentrations were recorded: spring (March to May), summer (June to August), autumn (September to November) or winter (December to February) [25]. Vitamin D supplementation and the use of carbamazepine, an anticonvulsant capable of reducing serum 25(OH)D concentrations, were also considered [32].

### 2.5. Statistical Analysis 

The sample characteristics according to serum 25(OH)D status at baseline were expressed as means, standard deviations, and proportions, and differences between groups were analyzed using the chi-square test and analysis of variance with Tukey’s post hoc test. Differences between included and excluded (due to the lack of information on serum 25(OH)D and covariates) individuals were analyzed using the chi-squared test and Student’s *t*-test.

Poisson regression models performed separately for men and women were adopted to verify the association between serum 25(OH)D status and the development of any difficulty in performing one or more IADL during the follow-up (incidence of IADL disability). Poisson regression is often used in longitudinal studies, where the outcome is the number of episodes of an event that occurred in a period of follow-up, and is more efficient than logistic regression in estimating the relative risk when the variable of interest is categorical [33].

For the regression models, covariates with *p*-value < 0.20 in the univariate analysis were selected for the multivariate models using the stepwise forward method. Statistical significance in the final model was considered by *p*-value < 0.05 [34]. In all analyses, the reference category for comparisons was the sufficient serum 25(OH)D (>50 nmol/L), and all analyses were performed in Stata 14 SE (StataCorp, College Station, TX, USA).

## 3. Results

### 3.1. Characteristics of Individuals at Baseline

Among the 4768 individuals at baseline, the mean age was 66 years, and the majority were women (53.1%), white, with upper wealth quintile, with conjugal life, former smokers, with frequent alcohol consumption and active lifestyle. The most prevalent health conditions were hypertension (34.8%), osteoarthritis (33.9%) and heart disease (14.4%). The prevalence of insufficient and deficient serum 25(OH)D concentrations was 32.3% and 22.9, respectively (Table 1 and Table 2).

Individuals with deficient serum 25(OH)D had lower wealth quintile, were more often smokers, drank rarely or never, and had a higher prevalence of hypertension, depression, and abdominal obesity than those with sufficient or insufficient serum 25(OH)D. Moreover, individuals with deficient serum 25(OH)D have a higher proportion of conjugal life, lower daily alcohol consumption, lower prevalence of osteoporosis, worse memory performance, a higher mean WC, and were more obese according to BMI compared to those with sufficient 25(OH)D (Table 1 and Table 2). The sample characteristics according to sex are shown in Table S1.

The comparisons between included and excluded individuals demonstrated that the excluded participants were older (69.6 ± 10.6), with a lower wealth quintile and schooling level, drank less and were more sedentary when compared to those included. Excluded individuals also had more hypertension, diabetes mellitus, cancer, stroke, worse memory performance, obesity and dynapenia in comparison to included individuals (Table S2).

### 3.2. Vitamin D Status and Risk of Incidence of IADL Disability

According to Poisson models, deficient serum 25(OH)D increased the risk of incidence of IADL disability by 43% in men (IRR: 1.43; 95% CI 1.02, 2.00 *p* = 0.039) compared to men with sufficient serum. However, no significant association was found for women (IRR: 1.23; 95% CI 0.94, 1.62; *p* = 0.125). The results for insufficient serum 25(OH)D were also not significant for both sexes (Table 3).

## 4. Discussion

Our findings showed that in English older adults aged ≥50 years, deficient serum 25(OH)D was an independent risk factor for the incidence of IADL disability only in men. 

Previous studies have confirmed a close relationship between low serum 25(OH)D concentrations and functional repercussions in older adults, such as disability [10,11]. Specifically on BADL, it is already established that deficient serum 25(OH)D increases the risk of incidence of disability [25,35,36]. Nevertheless, even with evidence of a cross-sectional association between low serum 25(OH)D concentrations and low IADL scores [17,18], longitudinal association between serum 25(OH)D concentrations and incident IADL disability remains rarely explored and with conflicting results.

Kotlarczyk and colleagues [19], in a study of 137 older women recruited from long-term care facilities, showed that women with deficient serum 25(OH)D (<50 nmol/L) at baseline had a greater decline in IADL score at 12 (−2.0 ± 0.4 *p* < 0.01) and 24 months (−2.5 ± 0.6 *p* < 0.01) in comparison to those with sufficient serum (−0.5 ± 0.3 *p* < 0.01 in 12 months and −1.2 ± 0.3, *p* < 0.01 in 24 months). In contrast, Hakeen and colleagues [20] followed 775 individuals aged ≥ 85 years for 5 years and showed in analysis stratified by sex that low serum 25(OH)D concentrations (≤25 nmol/L) were associated with mild to moderate disability in men (OR = 3.83; 95% CI 0.95–2.74), and with low to mild (OR = 1.95; 95% CI 1.02–3.72) and mild to moderate disability in women (OR = 2,70; 95% CI 1.16–6.27) in models controlled by living in an institution, season, cognitive status, BMI and vitamin D supplementation. However, there was a loss of statistical significance after adjustment for physical activity.

The discrepancy between the aforementioned results and our findings may be due to the different methodologies used. First, although we did not find a significant association for women, Kotlarczyk and colleagues [19] had a female sample with very old participants, from long-term care facilities, and with a previous history of falls, impaired mobility, multimorbidity and polypharmacy, making these women more subject to functional decline than the women in our study. Second, Hakeen and colleagues [20] assessed disability trajectories due to difficulty in performing BADL and IADL together. Given that IADL are more complex activities that require highly complex neuropsychological organization, and the fact that IADL disability precedes ABVD disability, it is ideal that these activities be evaluated separately so that the results are not underestimated [15,37]. In addition, a decrease in the level of physical activity is expected as age advances. Thus, in a sample of older adults aged 85 years, physical activity may have a greater effect on disability than serum 25(OH)D concentrations, justifying the loss of statistical significance after adjusting for this factor, in the models by Hakeen and colleagues [20]. And finally, none of them verified the association between serum 25(OH)D status and the incidence of IADL disability, since individuals who already had disability at baseline were not excluded.

The possible mechanism by which deficient serum 25(OH)D is associated with the incidence of IADL disability is its close relationship with MSK and the CNS. Reduction of serum 25(OH)D concentrations results in less activation of genomic and non-genomic muscle pathways, which promote less stimulation of transcription factors responsible for myogenesis, a reduction in Ca^2+^ influx and absorption by myocytes. These events favor muscle atrophy, especially of type II fibers, with a consequent reduction in neuromuscular strength [12]. Consequently, musculoskeletal function is compromised and progressively predisposes to the reduction of functional capacity, mobility limitations and development of disability [38].

Serum 25(OH)D also has neuroprotective properties, since it regulates Ca^2+^ homeostasis, maintains the integrity of nerve conduction, inhibits the proliferation of pro-inflammatory substances and reactive oxygen species, and increases the plasma concentration of β-amyloid and glutathione levels in the CNS [14]. Thus, with the decrease in serum 25(OH)D concentrations, the CNS is more susceptible to injuries and degenerative processes that can favor cognitive decline [39], which represents an important risk factor for the incidence of IADL disability.

The hypotheses that support the sex differences found in this study are the biological differences in the metabolism of MSK and CNS between men and women. Men have a more evident impairment in type II fibers than women [40], partly due to the reduction in testosterone levels with aging [41]. Since 25(OH)D appears to act on testosterone synthesis in men, and serum 25(OH)D deficiency is associated with lower testosterone [42], we believe this mechanism may enhance the process of muscle atrophy in men. Regarding the CNS, due to the protective effect that testosterone exerts on the CNS [43] and the fact that its synthesis is dependent on serum concentrations of 25(OH)D [44], we assume that it is possible that low 25(OH)D concentrations may reduce testosterone levels and favor earlier and more gradual cognitive decline in men than in women.

The main strength of this study is its large representative sample of English individuals aged ≥50 years, which made it possible to carry out analyses stratified by sex. In addition, our models were adjusted for important covariates, and the use of two waves provided a reasonably long follow-up time.

However, our study has some limitations that need to be reported. Self-reported disability assessment can bring some information bias. Nevertheless, the Lawton scale has international validity for assessing IADL. The inclusion of individuals exclusively residing in the community makes it impossible to estimate the results for those institutionalized, who have a higher prevalence of IADL disability. Losses are also an inevitable source of bias in longitudinal studies. The exclusion of individuals with lack of data may represent another potential source of bias, since those excluded had characteristics considered as important risk factors for disability. However, this exclusion did not prevent us from finding a significant association for men. The lack of nutrition data in our analyses did not allow us to assess the quality of nutritional status, which represents an intrinsic mechanism for maintaining functionality in older adults [3]. For the same reason, it was also not possible to investigate the dietary intake of vitamin D sources that may contribute to the maintenance of serum 25(OH)D concentrations. Finally, information on parathyroid hormone (PTH) and testosterone serum concentration was also not collected. Serum PTH concentrations are high in the presence of serum 25(OH)D deficiency, which characterizes secondary hyperparathyroidism, a condition associated with reduced strength that can compromise functionality [13], while testosterone is related to the preservation of musculoskeletal and cognitive function [41,43].

## 5. Conclusions

We found a sex difference in the association between deficient serum 25(OH)D and incidence of IADL disability, with significant results only for men. Since 25(OH)D deficiency is a modifiable condition, it must be investigated early to prevent difficulty in performing IADL and consequently the development of disability. More studies are needed to better understand the mechanisms involved in the sex differences found. It is also crucial to know the trajectories of functional decline as a function of serum 25(OH)D concentration to develop assertive strategies to prevent disability.

## Figures and Tables

**Table 1:** Socioeconomic characteristics and health-related behaviors of 4768 participants without IADL disability at baseline according to serum 25(OH)D status, ELSA Study (2012–2013).

	Total (*n* = 4768)	Serum 25-Hydroxyvitamin D Concentrations		
		>50 nmol/L (*n* = 2135)	>30 to ≤50 nmol/L (*n* = 1543)	≤30 nmol/L (*n* = 1090)
Age, years (SD)	66.0 ± 8.5	66.1 ± 8.2	66.1 ± 8.6	65.7 ± 9.0
50–59	24.8	23.1	24.1	29.1 **
60–69	42.6	44.4	42.5	39.4 *
70–79	25.8	26.5	26.1	23.9
80–89	6.4	5.7	7.0	7.1
≥90	0.4	0.3	0.3	0.5
Sex (women) %	53.1	52.5	51.4	56.7
Race (non-white) %	2.7	1.1	2.5 *	6.1 **
Conjugal life (yes) %	69.4	74.1	67.6 *	62.6 *
Schooling, %				
>13 years	35.4	36.4	35.7	33.0
12 to 13 years	28.9	28.9	29.6	27.8
≤11 years	35.7	34.7	34.7	39.2
Wealth, %				
Upper quintile	25.1	29.1	24.6 *	18.2 **
4° quintile	22.8	24.3	23.5	19.0 **
3° quintile	21.0	22.2	20.3	19.8
2° quintile	17.6	14.8	17.8	22.8 **
Lower quintile	11.4	7.9	11.4 *	18.1 **
Not applicable	2.1	1.7	2.4	2.1
Smoking, %				
Non-smoker	39.9	40.3	40.8	37.9
Former smoker	49.5	52.4	49.1	44.4 *
Current smoker	10.6	7.3	10.1 *	17.7 **
Alcohol intake, %				
Rarely/never	16.3	13.0	16.5 *	22.4 **
Frequently	40.8	41.6	42.1	37.5
Daily	35.6	40.5	33.6 *	29.1 *
Not applicable	7.3	4.9	7.8 *	11.0 *
Physical activity (sedentary), %	1.9	1.5	1.7	3.0

Data expressed as percentage, mean, and standard deviation (SD) values. * Significant difference from sufficient. ** Significant difference from sufficient and insufficient (*p* < 0.05).

**Table 2:** Health conditions, anthropometric measures, and covariates of 4768 participants without IADL disability at baseline according to serum 25(OH)D status, ELSA Study (2012–2013).

	Total (*n* = 4768)	Serum 25-Hydroxyvitamin D Concentrations		
		>50 nmol/L (*n* = 2135)	>30 to ≤50 nmol/L (*n* = 1543)	≤30 nmol/L (*n* = 1090)
Health conditions, %				
Hypertension	34.8	32.4	35.9	37.9 **
Diabetes mellitus	8.2	7.3	8.7	9.4
Cancer	2.5	5.5	3.9	4.2
Heart disease	13.6	13.7	13.8	13.0
Lung disease	11.9	11.6	11.8	12.6
Stroke	2.7	2.2	2.5	3.8
Osteoporosis	6.6	8.8	5.6 *	3.7 *
Osteoarthritis	33.9	33.5	33.4	35.4
Dementia	0.3	0.2	0.2	0.5
Falls	18.0	18.6	18.9	15.5
Depressive symptoms	8.3	6.7	7.8	12.2 **
Memory (SD)	11.3 ± 3.3	11.5 ± 3.3	11.2 ± 3.3	11.1 ± 3.7 *
Seasonality, %				
Spring	22.7	30.9	20.5 *	9.6 **
Summer	8.1	5.2	7.9 *	13.9 **
Autumn	42.5	46.2	44.8	32.2 **
Winter	26.7	17.7	26.8 *	44.3 **
Vitamin D supplementation, %	4.5	4.6	4.5	4.0
Use of carbamazepine, %	1.9	1.9	2.0	1.7
WC, cm (SD)	95.4 ± 18.6	93.7 ± 23.3	96.4 ± 13.2 *	97.3 ± 14.3 *
>102 men >88 women %	48.2	41.7	51.2 *	56.7 **
BMI, kg/m^2^ (SD)	27.8 ± 4.8	27.1 ± 4.3	28.2 ± 4.8 *	28.7 ± 5.4 *
≥18.5 and <25 kg/m^2^	28.3	32.3	25.4 *	24.4 *
<18.5 kg/m^2^	0.8	1.0	0.4	1.2
≥25 and <30 kg/m	43.3	45.3	43.7	39.0 *
≥30 kg/m	27.6	21.4	30.5 *	35.4 *
Grip strength, kg (SD)	32.0 ± 11.4	32.3 ± 11.3	32.2 ± 11.3	31.2 ± 11.4
<26 men <16 women %	6.2	5.9	6.2	7.0

Data expressed as percentage, mean, and standard deviation (SD) values. BMI: body mass index; WC: waist circumference. * Significant difference from sufficient. ** Significant difference from sufficient and insufficient (*p* < 0.05).

**Table 3:** Final adjusted Poisson regression models for incidence of IADL disability during a 4-year follow-up, ELSA Study (2012–2017).

25(OH)D Status	IRR for Incidence of Disability in IADL	
	Men ^1^ (*n* = 1887)	Women ^2^ (*n* = 2165)
Sufficient (>50 nmol/L)	1.00	1.00
Insufficient (>30 to ≤50 nmol/L)	1.02 [0.76–1.38]	1.19 [0.93–1.53]
Deficient (≤30 nmol/L)	1.43 [1.02–2.00]	1.23 [0.94–1.62]

Values presented by incidence rate ratio (IRR) and 95% confidence interval. 25(OH)D: 25-hydroxyvitamin D; IADL: instrumental activities of daily living. ^1^ Controlled by race, age, physical activity, depression symptoms, osteoarthritis, falls, schooling, smoking, cognition, osteoporosis, lung disease, season, use of carbamazepine and vitamin D supplementation. ^2^ Controlled by race, age, neuromuscular strength, physical activity, osteoarthritis, falls, depressive symptoms, hypertension, osteoporosis, schooling, smoking, waist circumference, lung disease, vitamin D supplementation, cognition, season, and use of carbamazepine.

## Data Availability

The English Longitudinal Study of Ageing data are available to the scientific community from the UK Data Service for researchers who meet the criteria for access to confidential data, under conditions of the End User License http://ukdataservice.ac.uk/media/455131/cd137-enduserlicence.pdf (accessed on 10 May 2022). The data can be accessed from: https://beta.ukdataservice.ac.uk/datacatalogue/series/series?id=200011#!/access-data (accessed on 10 May 2022). Contact with the UK Data Service regarding access to the English Longitudinal Study of Ageing can be made through the website https://www.ukdataservice.ac.uk/about-us/contact (accessed on 10 May 2022), by phone +44 (0)1206 872143, or by email at help@ukdataservice.ac.uk.

## Supplementary Materials

The following supporting information can be downloaded at: https://www.mdpi.com/article/10.3390/nu14102012/s1. Table S1: Characteristics of 4768 individuals free of IADL disability at baseline according to sex, ELSA Study (2012–2013). Table S2: Characteristics of individuals included and excluded due to lack of data on covariates, ELSA Study (2012–2013).
