# Supplementary material for: Sex Differences in Vitamin D Status as a Risk Factor for Incidence of Disability in Instrumental Activities of Daily Living: Evidence from the ELSA Cohort Study
Source: Nutrients. 2022 May 11;14(10):2012. doi: 10.3390/nu14102012 (PMC9145423; doi:10.3390/nu14102012)
Supplement: Supplementary file 1 [file nutrients-14-02012-s001.zip › nutrients-1692210-supplementary.pdf]

Table S1. Characteristics of 4.768 individuals free of IADL disability at baseline according to sex, ELSA Study (2012-2013).

	**Total (*n* = 4,768)**	**Men (*n* = 2,236)**	**Women (*n* = 2,532)**
**Age, years (SD)**	66.0 ± 8.5	66.1 ± 8.5	66.0 ± 8.5
50 – 59	24.8	24.6	25.0
60 – 69	42.6	42.9	42.4
70 – 79	25.8	25.3	26.2
80 – 89	6.4	6.8	6.1
≥ 90	0.4	0.4	0.3
**Race (non-white) %**	2.7	3.1	2.4
**Marital status (with conjugal life) %**	69.4	76.3*	63.3*
**Schooling, %**			
> 13 years	35.4	43.1*	28.6*
12 to 13 years	28.9	25.3*	32.0*
≤ 11 years	35.7	31.6*	39.4*
**Wealth, %**			
Upper quintile	25.1	26.7	23.8
4° quintile	22.8	23.8	22.0
3° quintile	21.0	20.9	21.1
2° quintile	17.6	16.5	18.6
Lower quintile	11.4	10.1	12.4
Not applicable	2.1	2.0	2.1
**Smoking, %**			
Nonsmoker	39.9	34.1*	45.1*
Former smoker	49.5	55.4*	44.2*
Smoker	10.6	10.5	10.7
**Alcohol intake, %**			
Rarely/never	16.3	11.1*	20.9*
Frequently	40.8	38.0*	43.4*
Daily	35.6	43.0*	29.1*
Not applicable	7.3	7.9	6.6
**Physical activity (sedentary) %**	1.9	2.0	1.9
**Health conditions, %**			
Hypertension	34.8	37.7*	32.3*
Diabetes mellitus	8.2	10.4*	6.3*
Cancer	2.5	2.8	2.3
Heart disease	13.6	16.0*	11.5*
Lung disease	11.9	11.3	12.4
Stroke	2.7	3.3	2.1
Osteoporosis	6.6	2.2*	10.4*
Osteoarthritis	33.9	27.6*	39.5*
Dementia	0.3	0.4	0.1
Falls	18.0	16.0*	19.8*
Depressive symptoms	8.3	6.3*	10.2*
Memory (SD)	11.3 ± 3.3	10.9 ± 3.2*	11.7 ± 3.4*
**Serum 25(OH)D, nmol/L (SD)**	49.8 ± 23.3	50.1 ± 23.0	49.5 ± 23.6
Sufficient (> 50 nmol/L)	44.8	45.4	44.3
Insufficient (> 30 to ≤ 50 nmol/L)	32.3	33.5	31.3
Deficient (≤ 30 nmol/L)	22.9	21.1	24.4
**Seasonality, %**			
Spring	22.7	22.2	23.1

Summer	8.1	7.7	8.4
Autumn	42.5	42.9	42.3
Winter	26.7	27.2	26.2
**Vitamin D supplementation, %**	4.5	4.7	4.2
**Use of carbamazepine, %**	1.9	1.8	1.9
**WC, cm (SD)**	95.4 ± 18.6	101.3 ± 22.0*	89.4 ± 12.4*
> 102 men > 88 women, %	48.2	42.7*	53.1*
**BMI, kg/m^2 (SD)**	27.8 ± 4.8	28.0 ± 4.2*	27.6 ± 5.2*
≥ 18.5 and < 25 kg/m^2	28.3	22.9*	32.9*
< 18.5 kg/m^2	0.8	0.3*	1.3*
≥ 25 and < 30 kg/m	43.3	50.0*	37.5*
≥ 30 kg/m	27.6	26.8	28.3
**Grip strength, kg (SD)**	32.0 ± 11.4	40.5 ± 9.6*	24.5 ± 6.5*
< 26 men < 16 women, %	6.2	5.3	7.1

Data expressed as percentage, mean, and standard deviation (SD) values. BMI: body mass index; WC: waist circumference. * Statistically significant difference between sexes ($P < 0.05$).

Table S2. Characteristics of individuals included and excluded due to lack of data on covariates, ELSA Study (2012-2013)

	Included (*n* = 4,768)	Excluded (*n* = 2,490)
**Age, years (SD)**	66.6 ± 8.9*	69.6 ± 10.6*
50 – 59	24.8	24.5
60 – 69	42.6*	36.5*
70 – 79	25.8	26.7
80 – 89	6.4*	10.7*
≥ 90	0.4*	1.6*
**Sex, (women) %**	53.1	54.2
**Race (non-white) %**	2.7*	4.6*
**Marital status (with conjugal life) %**	69.4	66.4
**Schooling**		
> 13 years	35.4*	31.1*
12 to 13 years	28.9	26.3
≤ 11 years	35.7*	42.6*
**Wealth, %**		
Upper quintile	25.1*	21.5*
4° quintile	22.8	21.1
3° quintile	21.0	20.7
2° quintile	17.6	17.7
Lower quintile	11.4*	16.5*
Not applicable	2.1	2.5
**Smoking, %**		
Nonsmoker	39.9	36.8
Former smoker	49.5	50.5
Smoker	10.6	12.7
**Alcohol intake, %**		
Rarely/never	16.3	17.7
Frequently	40.8*	33.0*
Daily	35.6*	27.6*
Not applicable	7.3*	21.7*
**Physical activity (sedentary) %**	1.9*	4.3*
**Health conditions (yes), %**		
Hypertension	34.8*	40.1*
Diabetes mellitus	8.2*	12.1*
Cancer	4.7*	7.0*
Heart disease	13.6*	20.4*
Lung disease	11.9	13.2
Stroke	2.7*	4.2*
Osteoporosis	6.6	5.9
Osteoarthritis	33.9	31.8
Dementia	2.3	0.1
Falls	18.0	17.7
Depressive symptoms	8.3	10.2
Memory (SD)	11.1 ± 3.4*	9.8 ± 4.02*
**Seasonality, %**		
Spring	22.7	18.0
Summer	8.1	4.8

Autumn	42.5	42.7
Winter	26.7*	34.5*
**Vitamin D supplementation, %**	4.5	4.3
**Use of carbamazepine, %**	1.9	2.1
**WC, cm (SD)**	95.9 ± 18.1	98.7 ± 25.2
>102 men > 88 women	48.2	57.4
BMI, kg/m^2	28.1 ± 5.0	29.1 ± 5.9
≥ 18.5 and < 25 kg/m^2, %	28.3*	24.0*
< 18.5 kg/m^2, %	0.8	0.9
≥ 25 and < 30 kg/m^2, %	43.3	40.5
≥ 30 kg/m^2, %	27.6*	34.6*
**Grip strength, kg**	30.9 ± 11.6	27.3 ± 12.5
< 26 men < 16 women, %	6.2*	11.3*

Notes: Data expressed as percentage, mean, and standard deviation (SD) values. BMI: body mass index; WC: waist circumference. * Statistically significant difference between included and excluded ($P < 0.05$).
